# Framework and baseline examination of the German National Cohort (NAKO)

**DOI:** 10.1007/s10654-022-00890-5

**Published:** 2022-10-19

**Authors:** Annette Peters, German National Cohort (NAKO) Consortium, Annette Peters, Karin Halina Greiser, Susanne Göttlicher, Wolfgang Ahrens, Maren Albrecht, Fabian Bamberg, Till Bärnighausen, Heiko Becher, Klaus Berger, Achim Beule, Heiner Boeing, Barbara Bohn, Kerstin Bohnert, Bettina Braun, Hermann Brenner, Robin Bülow, Stefanie Castell, Antje Damms-Machado, Marcus Dörr, Nina Ebert, Margit Ecker, Carina Emmel, Beate Fischer, Claus-Werner Franzke, Sylvia Gastell, Guido Giani, Matthias Günther, Kathrin Günther, Klaus-Peter Günther, Johannes Haerting, Ulrike Haug, Iris M. Heid, Margit Heier, Diana Heinemeyer, Thomas Hendel, Florian Herbolsheimer, Jochen Hirsch, Wolfgang Hoffmann, Bernd Holleczek, Heike Hölling, Andreas Hörlein, Karl-Heinz Jöckel, Rudolf Kaaks, André Karch, Stefan Karrasch, Nadja Kartschmit, Hans-Ulrich Kauczor, Thomas Keil, Yvonne Kemmling, Bianca Klee, Birgit Klüppelholz, Alexander Kluttig, Lisa Kofink, Anna Köttgen, Daniel Kraft, Gérard Krause, Lisa Kretz, Lilian Krist, Jan Kühnisch, Oliver Kuß, Nicole Legath, Anna-Therese Lehnich, Michael Leitzmann, Wolfgang Lieb, Jakob Linseisen, Markus Loeffler, Anke Macdonald, Klaus H. Maier-Hein, Nina Mangold, Claudia Meinke-Franze, Christa Meisinger, Juliane Melzer, Björn Mergarten, Karin B. Michels, Rafael Mikolajczyk, Susanne Moebus, Ulrich Mueller, Matthias Nauck, Thoralf Niendorf, Konstantin Nikolaou, Nadia Obi, Stefan Ostrzinski, Leo Panreck, Iris Pigeot, Tobias Pischon, Irene Pschibul-Thamm, Wolfgang Rathmann, Achim Reineke, Stefanie Roloff, Dan Rujescu, Stefan Rupf, Oliver Sander, Tamara Schikowski, Sabine Schipf, Peter Schirmacher, Christopher L. Schlett, Börge Schmidt, Georg Schmidt, Martin Schmidt, Gina Schöne, Holger Schulz, Matthias B. Schulze, Alexandra Schweig, Anja M. Sedlmeier, Sonja Selder, Julia Six-Merker, Ramona Sowade, Andreas Stang, Oliver Stegle, Karen Steindorf, Gunthard Stübs, Enno Swart, Henning Teismann, Inke Thiele, Sigrid Thierry, Marius Ueffing, Henry Völzke, Sabina Waniek, Andrea Weber, Nicole Werner, H.-Erich Wichmann, Stefan N. Willich, Kerstin Wirkner, Kathrin Wolf, Robert Wolff, Hajo Zeeb, Melanie Zinkhan, Johannes Zschocke

**Affiliations:** 1 Institute of Epidemiology, Helmholtz Zentrum München – German Research Center for Environmental Health (GmbH), Ingolstädter Landstr. 1, 85764 Neuherberg, Germany; 2 Chair of Epidemiology, Institute for Medical Information Processing, Biometry and Epidemiology, Medical Faculty, Ludwig-Maximilians-Universität München, Munich, Germany; 3 NAKO e.V., Am Taubenfeld 21/2, 69123 Heidelberg, Germany; 4 German Cancer Research Center in the Helmholtz Association DKFZ, Heidelberg, Germany; 5 Leibniz Institute for Prevention Research and Epidemiology – BIPS, Bremen, Germany; 6 Faculty 3: Mathematics and Computer Science, University of Bremen, Bibliothekstraße 1, 28359 Bremen, Germany; 7 Berufsgenossenschaft Nahrungsmittel und Gastgewerbe, Mannheim, Germany; 8 Department of Diagnostic and Interventional Radiology, Medical Center, Faculty of Medicine Freiburg, University of Freiburg, Freiburg, Germany; 9 Heidelberg University Hospital, Heidelberg, Germany; 10 University Medical Center Hamburg Eppendorf, Hamburg, Germany; 11 Institute of Epidemiology and Social Medicine, University of Münster, Münster, Germany; 12 Department of Otorhinolaryngology, University Hospital Münster, Münster, Germany; 13 Department of Otorhinolaryngology, Head and Neck Surgery, University Medicine Greifswald, Greifswald, Germany; 14 Department of Epidemiology (Closed), German Institute of Human Nutrition Potsdam Rehbruecke, Nuthetal, Germany; 15 Institute for Diagnostic Radiology and Neuroradiology, University Medicine Greifswald, Greifswald, Germany; 16 Helmholtz Center for Infection Research, Brunswick, Germany; 17 Department of Internal Medicine B, University Medicine Greifswald, Greifswald, Germany; 18 German Diabetes Center, Leibniz Center for Diabetes Research at Heinrich-Heine-University Düsseldorf, Düsseldorf, Germany; 19 Institute for Medical Informatics, Biometry and Epidemiology, Essen University Hospital, Essen, Germany; 20 University of Regensburg, Regensburg, Germany; 21 Institute for Prevention and Cancer Epidemiology, Faculty of Medicine and Medical Center, University of Freiburg, Freiburg, Germany; 22 German Institute of Human Nutrition Potsdam Rehbruecke, Nuthetal, Germany; 23 Fraunhofer Institute for Digital Medicine MEVIS, Bremen, Germany; 24 University Center of Orthopaedics, Traumatology and Plastic Surgery at the Technische Universität Dresden, Dresden, Germany; 25 Institute of Medical Epidemiology, Biostatistics, and Informatics, Medical Faculty of the Martin-Luther University Halle-Wittenberg, Halle, Germany; 26 Department of Genetic Epidemiology, University of Regensburg, Regensburg, Germany; 27 KORA Study Centre, University Hospital Augsburg, Augsburg, Germany; 28 Department of Radiology, Ludwig-Maximilians-University Hospital, Munich, Germany; 29 Institute for Community Medicine, University Medicine Greifswald, Greifswald, Germany; 30 Krebsregister Saarland, Saarbrücken, Germany; 31 Robert Koch-Institut, Berlin, Germany; 32 Institute and Clinic for Occupational, Social and Environmental Medicine, Ludwig-Maximilians-Universität München, Munich, Germany; 33 Institute of Social Medicine, Epidemiology and Health Economics, Charité – Universitätsmedizin Berlin, Berlin, Germany; 34 Institute of Clinical Epidemiology and Biometry, University of Würzburg, Würzburg, Germany; 35 Institute of Genetic Epidemiology, Faculty of Medicine and Medical Center – University of Freiburg, Freiburg, Germany; 36 Department of Conservative Dentistry and Periodontology, University Hospital, School of Dentistry, Ludwig-Maximilians-Universität München, Munich, Germany; 37 Institute of Epidemiology, University of Kiel, Kiel, Germany; 38 Helmholtz Zentrum München – German Research Center for Environmental Health (GmbH), Neuherberg, Germany; 39 Epidemiology, Medical Faculty, University of Augsburg, Augsburg, Germany; 40 Institute for Medical Information Processing, Biometry and Epidemiology, Medical Faculty, Ludwig-Maximilians University Munich, Munich, Germany; 41 University of Leipzig, Leipzig, Germany; 42 Pattern Analysis and Learning Group, Department of Radiation Oncology, Heidelberg University Hospital, Heidelberg, Germany; 43 Institute for Urban Public Health, Essen University Hospital, Essen, Germany; 44 Federal Institute for Population Research, Wiesbaden, Germany; 45 Institute of Clinical Chemistry and Laboratory Medicine, University Medicine Greifswald, Greifswald, Germany; 46 Max-Delbrueck Center for Molecular Medicine in the Helmholtz Association, Berlin, Germany; 47 Department of Diagnostic and Interventional Radiology, Eberhard Karls Universität Tübingen, Tübingen, Germany; 48 Molecular Epidemiology Research Group, Max Delbrück Center for Molecular Medicine in the Helmholtz Association (MDC), Berlin, Germany; 49 Biobank Technology Platform, Max Delbrück Center for Molecular Medicine in the Helmholtz Association (MDC), Berlin, Germany; 50 Hospital St. Marienstift Magdeburg GmbH, Magdeburg, Germany; 51 Medical University of Vienna, Vienna, Austria; 52 Chair of Synoptic Dentistry, Saarland University, Saarbrücken, Germany; 53 Department of Rheumatology, Heinrich-Heine-University Düsseldorf, Düsseldorf, Germany; 54 IUF – Leibniz Research Institute for Environmental Medicine, Duesseldorf, Germany; 55 Department of Internal Medicine 1, Klinikum rechts der Isar, Technical University Munich, Munich, Germany; 56 University of Potsdam, Potsdam, Germany; 57 Department of Ophthalmology, Tübingen University Hospital, Tübingen, Germany; 58 Institute of Social Medicine and Health Systems Research, Med. Faculty, Otto-Von-Guericke-University Magdeburg, Magdeburg, Germany; 59 University Hospital Augsburg, Augsburg, Germany; 60 Institute for Ophthalmic Research, Tübingen University Hospital, Tübingen, Germany; 61 Martin-Luther-University, Halle, Germany; 62 Institute of Physics, Martin-Luther University Halle-Wittenberg, Halle, Germany; 63 Charité – Universitätsmedizin Berlin, Berlin, Germany; 64 Germany State Institute of Health, Bavarian Health and Food Safety Authority, Erlangen, Germany

**Keywords:** Population-based cohort, Non-communicable diseases, Communicable diseases, Epidemiology, Life-style and socio-economic factors, Magnetic resonance imaging, Pre-clinical disease, Functional impairments, Psychosocial factors

## Abstract

The German National Cohort (NAKO) is a multidisciplinary, population-based prospective cohort study that aims to investigate the causes of widespread diseases, identify risk factors and improve early detection and prevention of disease. Specifically, NAKO is designed to identify novel and better characterize established risk and protection factors for the development of cardiovascular diseases, cancer, diabetes, neurodegenerative and psychiatric diseases, musculoskeletal diseases, respiratory and infectious diseases in a random sample of the general population. Between 2014 and 2019, a total of 205,415 men and women aged 19–74 years were recruited and examined in 18 study centres in Germany. The baseline assessment included a face-to-face interview, self-administered questionnaires and a wide range of biomedical examinations. Biomaterials were collected from all participants including serum, EDTA plasma, buffy coats, RNA and erythrocytes, urine, saliva, nasal swabs and stool. In 56,971 participants, an intensified examination programme was implemented. Whole-body 3T magnetic resonance imaging was performed in 30,861 participants on dedicated scanners. NAKO collects follow-up information on incident diseases through a combination of active follow-up using self-report via written questionnaires at 2–3 year intervals and passive follow-up via record linkages. All study participants are invited for re-examinations at the study centres in 4–5 year intervals. Thereby, longitudinal information on changes in risk factor profiles and in vascular, cardiac, metabolic, neurocognitive, pulmonary and sensory function is collected. NAKO is a major resource for population-based epidemiology to identify new and tailored strategies for early detection, prediction, prevention and treatment of major diseases for the next 30 years.

**Supplementary Information:**

The online version contains supplementary material available at 10.1007/s10654-022-00890-5.

## Introduction

Non-communicable diseases are considered the major threat for health worldwide [1]. Cancer and cardiovascular disease (CVD) remain the most important causes of death in Germany, accounting for 63% of all years of life lost in 2017 [2]. However, the past years have demonstrated that globally we face major challenges with immediate impacts and long-term consequences for health. These in particular include (1) the ageing population and the increase in social disparities, (2) the obesity epidemic, (3) the climate crisis, (4) the medical and public health impacts of the COVID-19 pandemic and (5) other emerging diseases. All five of these major challenges jointly change living conditions, environmental exposures, risk factor profiles, susceptibility and health service access.

Large-scale cohort studies are essential to understand the inherited and acquired determinants of health in populations and to shape the future of prevention and early disease detection. Furthermore, they provide us with insights about how living and working conditions of study participants change over time. Based on the advances in biomedical sciences, cohort studies are nowadays able to address the fundamental challenges of future health research in an unprecedented fashion by real-world assessments of population health and by generating and testing innovative hypotheses based on large-scale standardized observational data. Cohort studies are the prime source of inference in areas where randomized clinical trials are infeasible or unethical. Within the last 2 decades, a number of mega cohorts have been initiated worldwide to foster the understanding and prevention of non-communicable diseases [3–8]. Jointly with experimental and clinical studies, they guide novel approaches to personalized prevention, precision medicine and policies and population-based prevention to improve public health in a changing world.

The German National Cohort (NAKO, “NAKO Gesundheitsstudie”) is a large, multidisciplinary, prospective population-based cohort study [5, 9] (Table 1). The overarching scientific goals of NAKO are: (1) The identification of etiological pathways from life-style and environmental risk factors to major diseases and functional impairments. (2) The description and understanding of the causes of geographic and socio-economic disparities in health status and disease risks. (3) The development of risk prediction models for identifying individuals at increased risk for major diseases in a framework of personalized prevention strategies. (4) The evaluation of markers for early detection of disease and pre-disease phenotypes, in order to develop effective tools for disease prevention.

**Table 1:** Current and planned data collection in the German National Cohort

Data type	Number of participants	Details	Data acquisition period
Baseline assessment	Whole cohort: 205,415	Basic physical and medical examinations, face-to-face interview, touch-screen-based self-report questionnaires, biomaterial collections (Level 1)	2014–2019
Extended baseline assessment	56,971	Additional in-depth physical and medical examinations, additional self-administered questionnaires (touch-screen, teleform, web-based) (Level 2)	2014–2019
MRI	30,861	Whole-body 3T magnetic resonance imaging	2014–2019
Mortality follow-up	Whole cohort	Ascertainment of vital status, collection and coding of death certificates and clinical/forensic reports	2014–ongoing
Record linkage	Whole cohort	Statutory health insurances Private health insurances Epidemiological and clinical cancer registries Central Research Institute of Ambulatory Health Care in Germany German Statutory Pension Fund Institute for Employment Research	2014–ongoing
Calibration Study	5,903	Repeated extended examination with *Level 2* examination modules between 1 and 12 months after the baseline examination to assess short-term measurement variability	2016–2019
Written Health Follow-up questionnaire	Whole cohort	Self-reported incident events, with verification via medical records and questionnaires provided by treating physicians	2017–ongoing
First re-examination	135,000 planned	Face-to-face interview, touch-screen-based self-report questionnaires, repeat examinations, biomaterial collections	2018–2023
Supplementary COVID-19 questionnaire	160,227	Pandemic-related questions on general state of health, SARS-CoV-2 symptoms and tests, changes in behaviour and social contacts, psychological effects, changes in employment status, physical activity and consumption of stimulating substances	05–07/2020
SoccHealth	500 former professional soccer players (additional cohort)	Additional project assessing long-term effects of high-performance sports. Participants receive the *Level 2* programme and MRI of the first re-examination	2021–ongoing

NAKO is the largest epidemiologic study in Germany to date and a joint interdisciplinary endeavour of 27 German scientific institutions, including 15 universities, 4 Helmholtz health centres, 4 institutes of the Leibniz Association and 4 other national research institutions (see https://nako.de/allgemeines/der-verein-nako-e-v/organe-und-gremien/wissenschaftliche-projektleiter-der-mitgliedsinstitutionen/mitgliederversammlung/).

We report here on the NAKO baseline recruitment and assessment, and we describe the success and challenges in setting-up such a large population-based cohort as a national resource.

## Methods

### Study population and recruitment

**Fig. 1:**
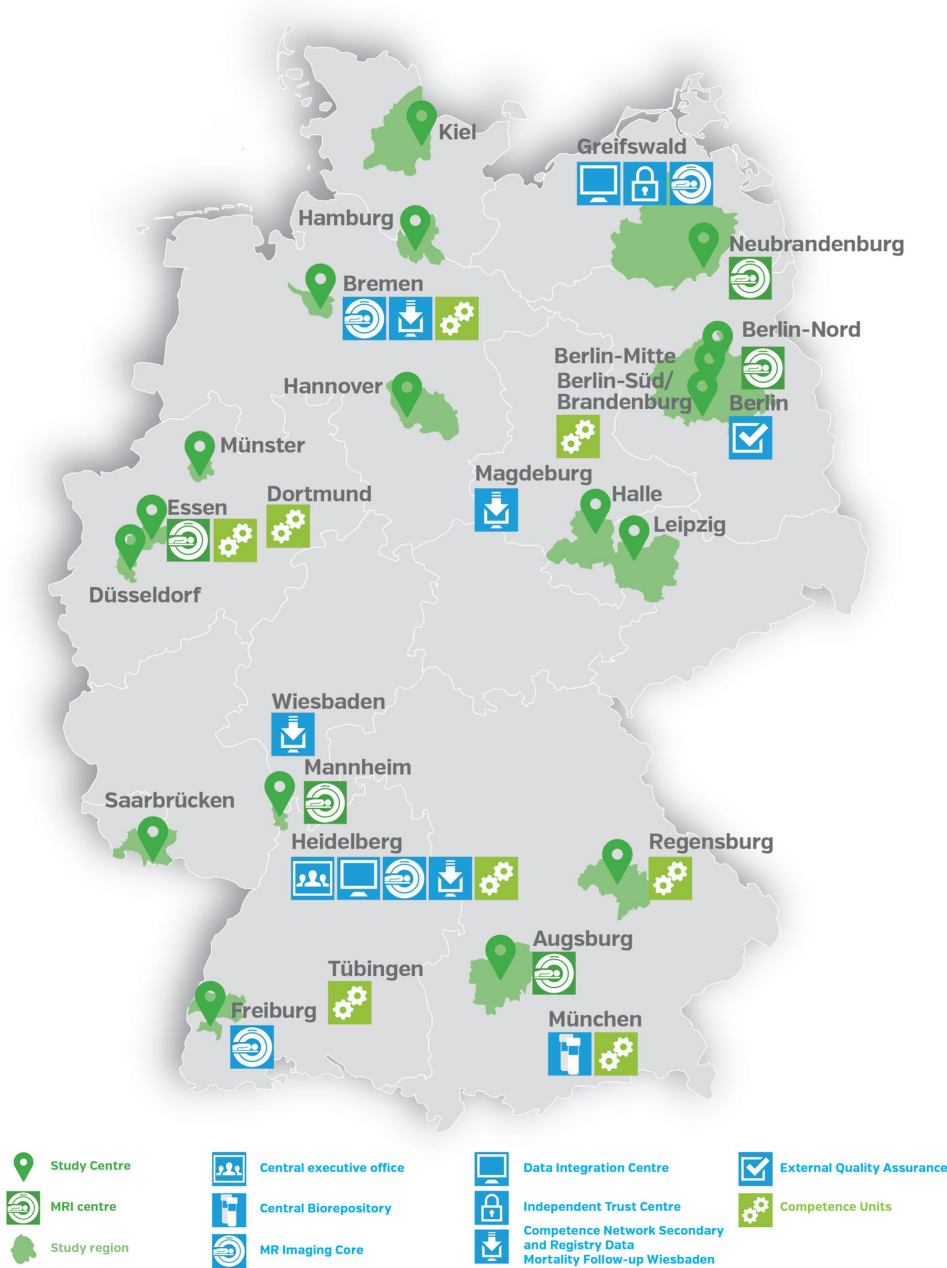
Study centres and infrastructures of the German National Cohort

NAKO set out to recruit a total of 200,000 residents aged 20 to 69 years at baseline within a 5-year period. Study participants were recruited through a network of 18 study centres from 16 study regions throughout Germany that include urban and industrialised areas as well as rural regions (Fig. 1). The goal was to recruit 10,000 participants each in 16 study centres and 20,000 participants each in 2 larger centres. All study participants were identified based on age and sex-stratified samples randomly drawn from compulsory registries of residents within the study areas. For both sexes, the design intended the overall recruitment of 10,000 participants in each 10-year age-group between 20 and 39 years, and 26,667 participants in each 10-year age-group between 40 and 69 years. The local study centres invited the participants for standardised assessments.

### Examinations and data collection

The baseline study programme included (1) a standardised, computer-assisted face-to-face interview, (2) biomedical examinations, (3) questionnaires to be filled in by the participants (mainly via touchscreen), (4) collection of biosamples, and (5) in 5 centres, a whole-body MRI of 30,000 participants. By design, data collection comprised two levels of intensity [5]. The standard *Level 1* programme was offered to all 200,000 participants and additional in-depth examinations to 20% randomly selected participants (*Level 2* programme). Magnetic resonance imaging (MRI) was planned to be performed in 30,000 participants. All MRI-participants were also invited to participate in the extended *Level 2* programme. An overview of the examination modules as part of the *Level 1* and *Level 2* programme is presented in Table 2. An overview of the 16 interview and the 30 touchscreen questionnaire modules is presented in Table 3.

**Table 2:** Disease groups and functions in focus of the baseline examination modules of the *Level 1* and *Level 2* programme

Disease group/function	Specific endpoints	Instruments (examinations in bold, technical devices used in italic)	
		Whole cohort (*Level 1*)	Subgroup (*Level 2*)
Cardiovascular Diseases	Myocardial infarction	Questionnaire	**10-s Electrocardiography** *(Cardio Perfect PRO, WelchAllyn, Skaneateles Falls, NY, USA)*
	Heart failure Diastolic/systolic dysfunction Valvular heart disease	Questionnaire	**3-D-echocardiography** *(Phillips iE33—Philips Medical Systems, Hamburg, Germany)* **MR Imaging of the heart** **Submaximal ergometry** [25] (Bicycle ergometer Sana Bike 350F—ergosana, Bitz, Germany; Heart rate chest strap T31—Polar Electro Oy, Finland; custom design ergometer software—Dr. Schmidt GmbH, Neunkirchen, Germany) (“1 of 3-exam”*)
	Hypertension/elevated blood pressure	Questionnaire **Blood pressure and heart rate** [26] *(HEM 705 IT -Omron Corporation, Kyoto, Japan)*	-
	Peripheral arterial disease Arterial stiffness	Questionnaire **Vascular Explorer** *(Enverdis, Düsseldorf, Deutschland)*	–
	Atrial fibrillation	Questionnaire	**10-s Electrocardiography** **Long-term electrocardiography** *(SOMNOwatch™ plus, SOMNOmedics; Randersacker, Germany)*
	Arteriovenous ratio of the retina	–	**Retinal photography** *(Retinal Camera CenterVue, DRS -Welch Allyn, Skaneateles Falls, NY, USA)*
Diabetes	Impaired glucose tolerance Diabetes mellitus	Questionnaire HbA1c **Oral glucose tolerance test** (subgroup of *Level 1*)	**Oral glucose tolerance test** (subgroup of *Level 2*) **Skin autofluorescence** (AGE reader SU—DiagnOptics Technologies, Groningen, The Netherlands)
	Diabetic retinopathy	–	**Retinal photography**
Cancer		Questionnaire	Tumor tissue bank
Neurologic and psychiatric diseases	Cerebrovascular diseases	Questionnaire	**MR Imaging of the brain**
	Cognitive impairment and dementia Impaired fine motor skills and coordination	Questionnaire **Neurocognitive test battery** [27]	**MR Imaging of the brain** **Number Series Test (touchscreen)** [28] **Purdue pegboard test**
	Depression Anxiety	Questionnaire	–
Respiratory diseases	Chronic obstructive pulmonary disease, asthma, lung function, airways inflammation	Questionnaire **Spirometry** [29] *(Easy on-PC spirometer—ndd Medizintechnik AG, Zürich, Switzerland)*	**Exhaled nitric oxide** (FeNO) [29] *(NIOX Vero—Circassia, Oxford, UK)*
Infectious disease	Selected acute transient infections	Questionnaire	–
	Gastrointestinal infections/intestinal microbiome	Questionnaire Stool samples (subgroup)	–
	Periodontal disease, craniomandibular disease, oral microbiome	Questionnaire **Tooth count** [30] Saliva sample	**Oral examination** (e. g., dental state, pocket depth, mandibular pain) [27] (custom design software—ParoStatus.de, Berlin, Germany) (“1 of 3-exam”)*
Sensory systems	Olfactory function	–	**Olfactory test** *(Sniffing Sticks Screening 12—Burghart Messtechnik, Holm, Germany)*
	Vision	–	**Visual acuity test** *(Testmonitor HP E241i (HP, Palo Alto, CA, USA)* **Retinal photography**
	Hearing		**Hearing test (German Digit Triplet Test)** [31, 32]
Musculoskeletal system	Arthrosis, rheumatoid arthritis	–	**Examination of knee and hip with angle chair** *(Orthopädie- und Rehatechnik Dresden, Dresden, Germany),* **Examination of hand** [33] (“1 of 3-exam”)*
Physical fitness and activity		Questionnaire **Hand grip strength** [25] *(Digital Dynamometer Jamar Plus*+ − *Sammons Preston, Rolyon, Bolingbrook, IL, USA)* **7-day accelerometry** [34] *(GT3X/*+ − *ActiGraph, Pensacola, FL, USA)*	**Ergometry** (“1 of 3-exam”)* **24 h accelerometry** (*SOMNOwatch™ plus, SOMNOmedics; Randersacker, Germany*)
Anthropometry [35]		**Height** *(SECA Stadiometer 274)* **Weight** *(SECA Medical Body Composition Analyzer mBCA 515)* **Waist circumference** *(SECA Circumference measuring tape 201)* **Body impedance/body fat** *(SECA Medical Body Composition Analyzer mBCA 515)* *All seca GmbH & Co. KG, Hamburg, Germany*	**Ultrasound of the abdominal fat** *(Phillips iE33—Philips Medical Systems, Hamburg, Germany)*

Modified and updated table based on [5] and [36]

*Each study centre selected one of three modules (1 of 3-exam system) to be carried out in all *Level 2* participants, while the other two modules were only performed in a subgroup of 100 participants. The angle chair and hand examinations counted as one 1 of 3 examination module

**Table 3:** Questionnaire data collected within the German National Cohort

Questionnaires
**Face to face interview**
*Level 1* mandatory modules (whole cohort)
Socio-economic status and socio-demographic factors: Nationality, ethnicity, native language, family status, housing situation, education, employment/profession/status, income, partners education, parents education, occupation
Medical history: Cardiovascular diseases, tumour diseases, metabolic diseases, musculoskeletal diseases, pulmonary diseases, allergies, gastrointestinal and liver diseases, skin diseases, kidney diseases, neurological and psychiatric diseases, eye, infectious diseases, other diseases (rheumatoid arthritis, morbus bechterew, lupus erythematodes, Sjögren syndrome, fibromyalgia, urinary tract stones, myelitis optic, multiple sclerosis, Tinnitus), operations; filter questions of Mini International Neuropsychiatric Interview vs 5.0, part Major Depression
Medication use (past 7 days)
Women: Menstrual cycle, contraceptives, menopause, hormone replacement, polycystic ovary syndrome, HPV
*Level 2* modules (subgroup)
If affirmative reply regarding depression in Mini International Neuropsychiatric Interview filter question: complete Mini questionnaire module on Major Depression
**Self-administered—touchscreen in study centre**
*Level 1* mandatory modules (whole cohort)
Health-related quality of life (modified SF-12)
Physical activity and fitness (VSAQ, Global Physical Activity Questionnaire)
Smoking
Alcohol consumption
Women: Pregnancies
Men: Family Planning
Infections and immune function
Participation in screening programmes
Pulmonary health
Oral health: Oral Health Impact Profile questionnaire (OHIP-5)
Cardiovascular health
Neurologic and psychiatric factors [37, 38]: depression (PHQ-9), anxiety (GAD-7)
Psychosocial factors [37]: Big-5 personality, stress (PHQ-Stress)
*Level 1* elective* modules (subgroup)
Instrumental Activities of Daily Living Lawton and Brody Questionnaire (age 60+ years)
Family history
Occupational reward/effort reward imbalance questionnaire (ERI)
Occupational exposures
Environmental exposures
Headache types/migraine
Drug use
Sleeping habits
Childhood/youth [39]
Childhood trauma questionnaire, short version (CT-S)
Social networks and support (adapted Berkman questionnaire) [37]
Weight history
Tattoos and solarium
Accidents and fractures
Gastrointestinal symptoms
Use of medical services
*Level 2* mandatory modules (subgroup)
Pain mannequin
Seeing and hearing
Restless Legs Syndrome
Exposure to animals
Work-related exposures/occupational stress (COPSOQ)
**Self-administered questionnaires at home**
Diet: Food Frequency Questionnaire (FFQ), short version 24 h recall: (sv24)
Physical activity [34]: Questionnaire on Annual Physical Activity Pattern (QUAP), computer-based 24-h physical activity recall (cpar24)

Modified and updated table based on [5]

*Level 1 elective program: for participants who did not complete the mandatory programme within 35 min the following rules applied: 50% receive the remaining modules in a random order, 50% in the fixed order. After 40 min, touchscreen ended for *Level 1* participants if they did not wish to complete all modules

All participants gave informed consent after receiving detailed information on the study content and procedures. Data collection was performed by specifically trained and certified study personnel. Biological samples of blood, urine, saliva, stool, and nasal swabs were obtained and processed on site.

Finally, a total of 30,000 participants were offered to undergo whole-body MRI using dedicated 3 Tesla scanners (Magnetom Skyra, Siemens Healthineers, Erlangen, Germany) at five MRI centres in Augsburg, Berlin, Essen, Mannheim and Neubrandenburg—(*MRI* programme) [10]. Identically installed MRI scanners remained technically (hard- and software) constant throughout the baseline recruitment period. The scanning protocol included sequences for the brain, the cardiovascular and musculoskeletal system as well as for the thorax and abdomen. Comprehensive measures assured homogeneous and highest quality of the acquired images. Moreover, procedures for the management of incidental findings were developed that included findings that were communicated to the study participants in order to inform about potential health problems that would require further medical attention [11].

A letter containing the basic results (e. a. blood parameters, blood pressure, anthropometric and accelerometry data) was sent to all study participants after the visit at the study centre.

### Central data management

Data were collected through standardised data entry forms and protocols for interviews and questionnaires as well as for all examinations at the study centres. For most medical devices, an automated transfer of examination data to the central data base was specifically programmed and implemented. Thus, all data were directly integrated in a central study database serviced at two data integration centres. This includes all data, also raw data collected during examinations by devices. These data integration centres are physically located at the University of Greifswald and at the German Cancer Research Center in the Helmholtz Association DKFZ, Heidelberg. The independent trust centre at the medical faculty of the University of Greifswald is responsible for personal identifying data storage, including addresses and consent management. For the MRI programme, incidental finding ascertainment and data storage infrastructure was built up [10].

### Collection and storage of biosamples

Whole blood, serum, EDTA plasma, erythrocytes, RNA, urine, saliva, nasal swabs and stool were collected from all study participants at baseline as part of the *Level 1* programme. The collection and local processing of the samples was highly standardised and includes the use of an automated liquid handling system for sample aliquoting [12]. More than two thirds of each individual’s aliquots collected during baseline recruitment are stored in a central biorepository at Helmholtz Munich [13] that is dedicated exclusively to NAKO. It includes – 80 °C semi-automated storage and − 180 °C storage in a fully automated sample handling robotic system for more than 20 million aliquots. One third of serum and plasma samples is stored at the local study centres for use in local analyses and as back-up storage.

### Follow-up

The follow-up of the cohort is essential to achieve the objectives of NAKO. Table 1 describes the approaches for data collection on incident events building upon the baseline recruitment described in this paper. All participants are followed via postal questionnaires (active follow-up), with the first 3-year follow-up initiated in 2017, and via record linkage with secondary data sources such as cancer registries and health insurance records (passive follow-up). All study population deaths are monitored from two main sources: notifications from study centres and regular vital status screening in German and other European residential registries. For all deaths, information on the exact date and place is collected, the death certificate plus medical and forensic reports accessed and coded in the ICD-10 framework in three versions: (1) as documented by the examining physician, (2) eventually re-arranged by the internationally established coding software IRIS, and (3) augmented by medical and forensic reports.

### Quality management

All data collection and biomedical examinations used standardised instruments and followed the procedures as described in specific standard operating procedure manuals (SOP). The study personnel underwent extensive training and was certified for examinations and interviews. Repeated centralised training and recertification took place at regular intervals. Comprehensive quality management included internal quality management organised by the central executive office and the study centres, and external quality management conducted by the Robert Koch Institute, Berlin.

### Data protection and ethics

Personal data are processed according to the concept on data privacy protection and IT security developed for the NAKO (see NAKO Gesundheitsstudie—Datenschutz in der NAKO). Essential principles of data protection addressed are (1) the separation of identifying data from other personal data, (2) the participants’ right of self-determination and control of own personal data, and (3) data reduction and data economy.

Adherence to legal requirements and to generally accepted ethical rules are fundamental principles for the conduct of NAKO. Structures, documents, and processes to ensure compliance of the conduct of the study with the ethical principles have been implemented. The ‘Code of Ethics’ of NAKO describes general ethical rules and principles for collection and use of study data (see NAKO Gesundheitsstudie—Ethik in der NAKO). An external ethics advisory board consists of members who represent ethical, social, scientific, medical, and legitimate matters in the area of life sciences and the study participants themselves. All study documents, including study protocols, participant information documents and declaration of consent forms for the baseline including the MRI examinations have been approved by all responsible local ethical committees. All ethics-related documents and processes are revised regularly and adapted as needed.

A modular consent declaration consisting of separate sections for participation in the main examination programme as well as specific programme modules, collection and storage of biosamples, retrieval of secondary data and repeated contact is applied to document the participants consent in detail (see NAKO Gesundheitsstudie—Einwilligung). Processes for collecting and documenting informed consent, for data processing consistent with the given consent and for handling of consent withdrawal have been implemented.

The linkage to external registries is ad persona. It can only be done for individuals who gave the specific informed consent. Linkage to health and pension insurances is done via the health insurance and pension insurance numbers, given by the participants during the consent process. Linkage to other registries is done via name, date of birth and address. To ensure data security and participant privacy, the linkage process is solely operationalized via the independent trust centre. Secondary data is retransmitted by the providers to the data integration centres only in pseudonymized form. These raw data are exclusively available to the competence network secondary and registry data of the NAKO, which is responsible for processing and coarsening. Only these processed data is incorporated into the research database and available for scientific analysis.

### Use and access

Access to and use of NAKO data and biosamples is regulated on the basis of a Use and Access Policy adopted by the General Assembly of NAKO (NAKO-e.-V._Nutzungsordnung_v2_2019-03-21.pdf) and is binding for all users. A transfer unit is responsible for the technical and administrative tasks related to the use and access procedures. Below, we briefly describe the current procedures. An electronic application portal (https://transfer.nako.de) was developed to support the applications for the use of NAKO data and in a separate modality for NAKO biosamples. A Use and Access Committee (UAC) evaluates the applications. The UAC's recommendation to accept or reject the application is presented to the members of the NAKO for potential objections. If no objection is raised, NAKO decides on the acceptance or rejection of the application. For biosamples, appropriateness of the proposed methodologies and the prioritisation given the limited amount of biomaterial is assessed in addition. After a data usage agreement has been signed by all involved institutions, the transfer unit provides the applicant with the data for analysis and supports the transfer of biosamples. Adherence to the EU-General Data Protection Regulation (GDPR) provisions is mandatory for all users.

## Results

### Recruitment, age distribution and response rates

The study centres started baseline recruitment between March and September 2014 and completed it between October 2018 and September 2019. Overall, 205,415 participants—out of which 59,971 with *Level 2* examinations and 30,861 with *MRI* examinations*—*were recruited, clearly exceeding the originally planned targets for the baseline examination. *Level 2* examinations were offered to *Level 1* participants, who had not been drawn into the random sample of *Level 2* participants, if they received the MRI examination.

**Fig. 2:**
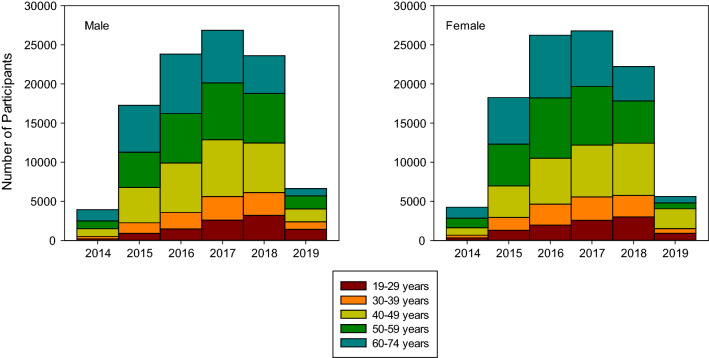
Age distribution of NAKO for men and women. N = 205,053 after excluding 362 participants who withdrew their consent until October 2019

The participants’ age at the day of the examination ranged from 19 to 74 years. By the end of the baseline recruitment, 362 participants (0.18%) had revoked their informed consent completely, resulting in 205,053 participants as of October 2019. Recruitment complied with the planned age-sex stratification of the cohort (Table 4). This was achieved by continuous monitoring of the age and sex distribution and intensified efforts to recruit within the age range 20–50 years towards the end (Fig. 2). The cohort shows a well-balanced age-sex distribution.

**Table 4:** Number of recruited NAKO participants by sex and age groups between 2014 and 2019. N = 205,053 after excluding 362 participants who withdrew their consent until October 2019

Study centre	Time period of recruitment	Sex		Age group					Total
		Female	Male	20–29	30–39	40–49	50–59	60+	
Augsburg	06/2014–09/2019	10,172	10,437	2,034	2,081	5,453	5,500	5,541	20,609
Berlin-Mitte	04/2014–01/2019	5,428	5,601	1,133	1,109	3,006	2,963	2,818	11,029
Berlin-Nord	06/2014–04/2019	5,134	4,960	986	1,051	2,629	2,731	2,697	10,094
Berlin-Sued	09/2014–04/2019	5,232	4,776	1,008	997	2,688	2,583	2,732	10,008
Bremen	03/2014–11/2018	5,318	5,171	1,083	1,161	2,679	2,794	2,772	10,489
Duesseldorf	06/2014–06/2019	4,684	4,450	978	958	2,305	2,471	2,422	9,134
Essen	03/2014–09/2019	5,336	5,316	1,118	1,288	2,782	2,756	2,708	10,652
Freiburg	06/2014–04/2019	5,032	5,049	995	1,030	2,668	2,698	2,690	10,081
Halle	05/2014–03/2019	5,247	4,881	939	967	2,585	2,821	2,816	10,128
Hamburg	09/2014–07/2019	5,160	4,927	992	1,071	2,599	2,738	2,687	10,087
Hannover	03/2014–11/2018	5,162	4,878	968	956	2,543	2,666	2,907	10,040
Kiel	04/2014–06/2019	4,866	4,628	925	1,042	2,422	2,578	2,527	9,494
Leipzig	08/2014–10/2018	5,413	5,443	1,091	1,154	2,894	2,873	2,844	10,856
Mannheim	05/2014–09/2019	5,182	5,108	1,069	1,102	2,699	2,720	2,700	10,290
Muenster	05/2014–06/2019	5,027	4,988	981	998	2,661	2,686	2,689	10,015
Neubrandenburg	03/2014–02/2019	10,917	11,079	1,837	2,577	5,272	6,086	6,224	21,996
Regensburg	04/2014–05/2019	4,992	5,032	951	995	2,673	2,720	2,685	10,024
Saarbruecken	06/2014–04/2019	5,169	4,858	899	1,141	2,574	2,625	2,788	10,027
Total		103,471	101,582	19,987	21,678	53,132	55,009	55,247	205,053
Proportion (%)		50	50	10	11	26	27	27

The overall response at baseline for NAKO was 17%. The response varied between 9 and 32% across study centres.

### Baseline examination and MRI

*Level 1* participants spent on average 215 min (3.6 h) at the study centre compared to 203 min planned. *Level 2* participants spent 340 min (5.6 h) compared to 306 min planned. Of this, examination and interview time amounted to about 155 min for the *Level 1* programme (of which ~ 30 min for interview and ~ 125 min for examinations), and 280 min for the *Level 2* programme (of which ~ 30 min for interview and ~ 250 min for examinations), and additional 15 min for informed consent ascertainment. The self-administered touchscreen questionnaires took ~ 45 min if all modules were completed.

*Level 1* participants completed 16 out of 16 interview modules. 94% of the *Level 1* participants completed all 8 examination modules and 89% all 30 touchscreen modules. *Level 2* participants completed in addition on average 8 out of 10 further examination modules and 79% of them all 10 modules. Table 5 lists the performed examinations and the percentage of participants who actually received the respective examination modules as compared to the originally planned target. Down time of measurement equipment and data capturing procedures, sick leaves of certified examiners and staff turnover in the study centres were main reasons for reduced completeness of the examination modules.

**Table 5:** Number of *Level 1* and the *Level 2* examination modules in 205,415 NAKO participants and completeness at baseline 2014–2019

	Modules	Number of participants	Completeness of modules (%)
Level 1	7-day accelerometry*	89,949	121
	Anthropometry	202,617	99
	Blood pressure	204,162	100
	Cognitive function	196,146	96
	Hand grip strength	201,972	98
	Spirometry	189,583	92
	Tooth count	189,086	92
	Vascular Explorer	194,589	95
Level 2**	3D echocardiography	41,040	72
	AGE Reader	46,585	82
	Electrocardiography	50,817	89
	Nitric oxide in exhaled air (FeNO)	41,895	73
	Olfactory test	54,827	96
	Purdue pegboard test	57,209	100
	Retinal photography	49,193	86
	SOMNOwatch	17,654	31
	Visual acuity test	51,364	90
	Ultrasound of visceral and subcutaneous abdominal fat	47,919	84
1 of 3 system***	Angle chair	22,002	89
	Examination of hand	23,306	94
	Ergometry	11,186	79
	Oral examination	20,814	98
Biosamples***	Blood	203,193	99
	Nasal swab	192,519	94
	Saliva	191,132	93
	Stool****	75,242	73
	Urine	200,927	98
	Oral glucose tolerance test	19,723	88

*7-day accelerometry was assigned to 50% of all participants who received only the *Level 1* examination programme, but was offered more frequently at some study centres

**56,971 participants received the *Level 2* programme

***Each study centre selected one of three modules (1 of 3 exam system) to be carried out in all *Level* 2 participants, while the other two modules are only performed in a subgroup of 100 participants. The angle chair and hand examinations counted as one 1 of 3 examination module. Some study centres chose 2 out of 3 modules and carried out each module on one-half of all *Level 2* participants

****Stool sample collection was assigned to 50% of all participants and started November 8th 2016

Overall, 30,861 participants underwent the whole-body MRI protocol at one of the five MRI centres, which was completely acquired with all imaging sequences in 94% of subjects. While MRI-centres in Augsburg and Neubrandenburg examined local study participants, participants from adjacent study centres were invited for participation in MRI-centres in Berlin-Nord, Essen and Mannheim. As such, at the MRI-centre located in Berlin-Nord, 3,878 examined participants were from Berlin-Mitte and Berlin Sued, at the MRI-centre in Essen 1,277 participants were from Muenster and Duesseldorf, and at the MRI-centre in Mannheim, 750 participants were from Saarbruecken und Freiburg.

### Biosample collection

The study centres processed and immediately froze at − 80 °C more than 19 million biosample aliquots as part of the baseline examinations. These were collected with a high degree of completeness. Per participant, 30 serum aliquots (à 0.25 mL; 99% completeness), 48 EDTA plasma aliquots (à 0.25 mL; 98% completeness), and 12 urine aliquots (à 0.25 mL; 98% completeness) were collected.

## Discussion

NAKO is a large prospective central European cohort of young, middle-aged and older women and men living within urban and rural regions of Germany. NAKO achieved its goal to recruit the planned number of participants within 5 years. In addition, NAKO also adhered to the planned sex and age distribution. This was possible through dedicated efforts. Participants with a migration background are well represented and comprised 16% within the first 100,000 participants [14]. Even though the participants with migration backgrounds are a very diverse group overall, subgroups of migrants can be studied separately with respect to region of origin due to the size of NAKO. The response proportion were substantially lower than the anticipated 50% at the planning stage, and we observed a considerable variation between the study centres. We consider a number of factors that may be responsible, including the urbanisation of the study region, the population composition in terms of migration background and education, and differences in local recruitment measures and strategies. We will assess the response proportions and regional differences in forthcoming publications.

NAKO was able to achieve a high degree of completeness for many of the examinations. Thus, an exceptional and rich database was created. The study is thereby a prime example for deep phenotyping in a large population-based cohort. The MRI scans provide a wealth of novel information to detect early changes within deeply phenotyped individuals. Jointly, it will allow assessing the role of early changes in function for the prediction of diseases and to derive signs of multimorbidity long before definite diagnoses are manifest. Quality assurance building on longstanding experience [15] as well as novel approaches employing machine learning and artificial intelligence techniques are underway to enrich the raw data by derived variables.

The data is complemented by a rich set of biosamples allowing for future analyses using targeted and untargeted high-throughput approaches as well as traditional laboratory analyses. The instant local processing combined with the complete cooling chain and the storage of blood and urine samples at − 180 °C will enable NAKO to provide high quality biosamples throughout the next decades. Characterisation of inherited and acquired molecular traits and their regulation is the cornerstone of innovative approaches to personalised prevention and medicine [16]. Understanding the role of genetics and molecular phenotypes such as transcriptome, proteome, metabolome, microbiome and other biomarkers can reveal new insights into the physiology of mechanisms as well as pathophysiology of disease development and progression. Life-style and environmental exposures determine and modify molecular phenotypes and form the basis for their impact on disease development [17]. The high quality of biosamples collected during the baseline examination and the subsequent data collections of NAKO provide an immense potential for implementation of well-established and novel high-throughput omics technologies. For example, we consider genotyping and whole genome sequencing, genome-wide methylation, RNA sequencing, metabolomics, proteomics, and serolomics. The NAKO biosampling is also remarkable due to its collection of samples for assessing the microbiome and virome of the gut, nose and oral cavity.

### Follow-up

An important design aspect of NAKO is that all participants are re-invited every 5 years to the study centres for repeated examinations. The extensive assessment by functional examinations permits the analysis of continuous changes over time, e.g., regarding vascular, cardiac, metabolic, neurocognitive, pulmonary and sensory function, but also in terms of changes in exposures. NAKO is currently inviting all participants to the first re-examination, which started between November 2018 and January 2020. The first re-examination was planned to be completed by April 2023; however, due to the COVID-19 pandemic, participation rates were lower than planned and temporary closures of study centres were unavoidable. Nevertheless, 66,870 study participants were re-examined (66% of forecasted numbers) including 9,889 MRI examinations (55% of forecasted numbers) until December 2021.

A further exceptional aspect is the platform-building feature of NAKO that enables ad hoc surveys in a well-defined group to answer imminent public health questions. For example, a supplementary COVID-19 questionnaire on SARS-CoV-2 infections and pandemic-related topics was sent out via email or letter to all study participants between April 30th and June 30th 2020. Overall, 160,227 questionnaires were completed, resulting in a response of 80.6%. While infection rates were very low during the first wave of the COVID-19 pandemic, clear increases in depression, anxiety and stress scores as compared to baseline levels were documented, in particular affecting the younger adults [18].

### Strengths and challenges

NAKO is characterized by a number of important design aspects that distinguish it from other international cohort studies and enables large-scale observational health research in Germany. NAKO is the largest cohort and harbours the largest biobank in Germany to date and is one of the largest in Europe. When compared to other large-scale epidemiological endeavours in Europe, NAKO has a substantially wider scope and assesses lifestyle characteristics and other exposures not only at baseline, but repeatedly and with innovative approaches. For example, the European Prospective Investigation into Cancer and Nutrition (EPIC) assessed lifestyle characteristics and anthropometric variables only once at baseline and did not include innovative tests and examinations like NAKO; disease follow-up in EPIC differs by country but is on a European level largely focussed on linkage with cancer and mortality registries (https://epic.iarc.fr/). Many other large cohorts are restricted to questionnaires and did not include any phenotyping in person (http://www.millionwomenstudy.org/; https://nurseshealthstudy.org/;). In NAKO, (i) the focus on repeated deep phenotyping will allow assessing the development of major chronic diseases and multimorbidity in an ageing society based on changes in risk factors and intermediate phenotypes of multiple diseases. (ii) The large number of participants below 40 years of age at the baseline examination (N = 41,642) is exceptional in comparison to cohorts internationally. (iii) NAKO collects and stores high quality biosamples repeatedly, which will allow innovative genomic analyses and application of omic-technologies repeatedly within the same individual. (iv) Repeated MRI scanning will allow characterizing changes over time with unprecedented depth. (v) The record linkage with secondary data sources including health insurance data, cancer registries, pension funds and the statutory occupational insurance will enrich the primary data. Thus, supplemental information on disease incidence, medical treatments and occupational histories will be ascertained which would be otherwise not available. (vi) NAKO will provide unique data for assessing the medical and public health impact of the COVID-19 pandemic building upon examination data collected before, during and after the pandemic.

There are, however, also a number of weaknesses. They include the low response proportions and therefore a possible reduced generalizability when it comes to estimation of risk factor distributions or disease or prevalence within the German population. Also, while the completeness of the modules overall is excellent, missingness due to different combinations of examination modules might pose a problem for certain analyses. Specific research questions may need to build on smaller samples sizes and consider the patterns of missingness.

One special element of NAKO is the fact that virtually the whole German epidemiological community has collaborated in the design and preparation of the cohort, and many of them are directly involved in the field work. Thus, NAKO constitutes an extraordinary basis for scientific cooperation and networking amongst epidemiologists and other health scientists in Germany. However, coordinating such a large and complex project jointly within the framework of the NAKO e.V. and collecting data at 18 study centres poses a number of challenges. Among them are (1) the consistent standardized data collection with high quality in 18 study centres simultaneously and over more than a decade, (2) the regional differences in institutional settings and state-based regulations including data protection, but also on other issues, such as COVID-19 abatement measures, (3) the different response rates between the centres, (4) quality assurance procedures involving more than 100 experts from the epidemiological community, (5) the complexity of data integration and synchronization of data availability.

Other methodological challenges arise from the underlying sampling frame of the study population, the varying response proportions, the complexity of the data, and the potential non-random structures in missing data. These aspects are addressed in more detail in Kuss et al. [19], providing guidance on data analyses strategies. The large-scale data will allow applying causal inference frameworks [20] as well as developing and testing approaches for best-practice of data analyses [21, 22].

### Outlook

Largescale cohort studies such as the UK Biobank show the immense potential to support biomedical discoveries and novel therapeutic approaches [23]. In addition, cohort studies are also prime resources to understand the health impacts in a rapidly changing world. The repeated survey of psychosocial, socioeconomic and behavioural factors over time will permit the analysis of the effect of environmental and societal changes and restrictions on health outcomes and wellbeing. Importantly, this will also provide insights into health impacts resulting from the COVID-19 pandemic including infections and containment measures. The NAKO study regions include urban and rural areas capturing diverse central European environmental conditions. Adding complex time and space resolved environmental exposure data will offer the opportunity to study the health impacts of climate change [24] and the benefits of climate change mitigation efforts on health within the next decades. The age-composition of the cohort provides longitudinal data on persons who will be up to 80 years old during the next years. We will therefore be able to provide data on factors relevant for healthy ageing and an ageing population. It also includes participants aged 20 years who can be followed in their health course for decades. The rich phenotyping data on subclinical findings and function will permit insights into trajectories of health and disease including multimorbidity. Thereby, NAKO will provide foundations for tailored recommendations on health and disease management strategies and form an exceptionally strong basis for designing interventional studies to promote healthy ageing and to slow down progression to overt disease.

## Conclusion

NAKO provides a central platform for future epidemiological research with a strong potential to push the development of new strategies for prevention, early detection and risk stratification of major chronic diseases. With its broad spectrum of examinations, the systematic re-assessments of all study participants and its high-quality biomaterials, NAKO provides an excellent tool for future, population-based longitudinal research. The large-scale, embedded MRI programme is another asset of NAKO. The German National Cohort, NAKO, thus constitutes an extraordinary basis for scientific cooperation and networking amongst epidemiologists internationally.

## Supplementary information

The German National Cohort (NAKO) Consortium consists of persons responsible for the planning and execution of the study, namely PIs and Co-PIs, the heads of study centres, infrastructures and competence units and the persons responsible for the study modules – both current and former, if they were active during baseline. An overview of the people contributing to NAKO can be found in the Supplementary Tables 1 to 5.

## Supplementary Information

Below is the link to the electronic supplementary material.

Supplementary file1 (XLSX 23 kb)

## Data Availability

Access to and use of NAKO data and biosamples can be obtained via an electronic application portal (https://transfer.nako.de).
